# Comprehensive fragmentation of cell-free repetitive DNA for enhanced cancer detection in plasma

**DOI:** 10.3389/fcell.2025.1630231

**Published:** 2025-07-09

**Authors:** Mingguang Zhang, Shuohui Dong, Wei Rao, Shiwen Mei, Gang Hu, Ling Liu, Zhen Wang, Jianqiang Tang

**Affiliations:** ^1^ Department of Colorectal Surgery, National Cancer Center/National Clinical Research Center for Cancer/Cancer Hospital, Chinese Academy of Medical Sciences and Peking Union Medical College, Beijing, China; ^2^ Department of General Surgery, Qilu Hospital of Shandong University, Jinan, China; ^3^ Department of Pathology, National Cancer Center/National Clinical Research Center for Cancer/Cancer Hospital, Chinese Academy of Medical Sciences and Peking Union Medical College, Beijing, China; ^4^ College of Bioinformatics Science and Technology, Harbin Medical University, Harbin, Heilongjiang, China; ^5^ Department of Thoracic Surgery, National Cancer Center/National Clinical Research Center for Cancer/Cancer Hospital, Chinese Academy of Medical Sciences and Peking Union Medical College, Beijing, China

**Keywords:** cell-free DNA, repetitive element, early tumor detection, tissue of origin, low-pass whole genome sequencing

## Abstract

**Background:**

Repetitive elements account for a large proportion of the human genome and undergo alterations during early tumorigenesis. However, the exclusive fragmentation pattern of DNA-derived cell-free repetitive elements (cfREs) remains unclear.

**Methods:**

This study enrolled 32 healthy volunteers and 112 patients with five types of cancer. A novel repetitive fragmentomics approach was proposed to profile cfREs using low-pass whole genome sequencing (WGS). Five innovative repetitive fragmentomic features were designed: fragment ratio, fragment length, fragment distribution, fragment complexity, and fragment expansion. A machine learning-based multimodal model was developed using these features.

**Results:**

The multimodal model achieved high prediction performance for early tumor detection, even at ultra-low sequencing depths (0.1×, AUC = 0.9824). Alu and short tandem repeat (STR) were identified as the primary cfREs after filtering out low-efficiency subfamilies. Characterization of cfREs within tumor-specific regulatory regions enabled accurate tissue-of-origin (TOO) prediction (0.1×, accuracy = 0.8286) and identified aberrantly transcribed tumor driver genes.

**Conclusion:**

This study highlights the abundance of repetitive DNA in plasma. The innovative fragmentomics approach provides a sensitive, robust, and cost-effective method for early tumor detection and localization.

## 1 Introduction

Cell-free DNA (cfDNA) consists of DNA fragments released during cell death and subsequently degraded by nucleases. It functions as a non-invasive biomarker for the detection of cancer. The fragmentation pattern of cfDNA exhibits a non-random distribution throughout the genome, aiding in the identification of early-stage cancer patients from healthy individuals (Lo et al., 2021; Snyder et al., 2016; Cristiano et al., 2019; Chabon et al., 2020; Ulz et al., 2016). Previous studies have identified certain characteristic features of cfDNA fragments, including lengths of approximately 166∼167 bp and peaks at 10 bp intervals, which correlate with the distribution of nucleosomes (Snyder et al., 2016). Additionally, the various nucleases and nucleosome accessibility can result in preferential fragment shearing positions, producing different patterns of fragment end motifs (Serpas et al., 2019; Jiang et al., 2020). In recent years, several studies have made progress in the use of cfDNA fragmentomics for early cancer detection. However, each approach has its own limitations (Cristiano et al., 2019; Ulz et al., 2016; Jiang et al., 2020; Jiang et al., 2015; Sun et al., 2019; Ulz et al., 2019). For instance, the utilization of genome-wide biomarkers, such as fragment length ratios (DELFI) (Cristiano et al., 2019), genome instability based on fragment coverage (CIN) (Jiang et al., 2015), and diversity scores of fragment end motifs (MDS) (Jiang et al., 2020), fails to elucidate the relationship between genes and transcriptional regulation. Furthermore, the accuracy of the calculated biomarkers in distinguishing cancer patients from healthy individuals based on fragment depth and various functional elements, such as transcription factor binding sites (TFBSs) (Ulz et al., 2019) and transcription start sites (TSSs) (Ulz et al., 2016), is limited. Therefore, these challenges constrain the clinical application and dissemination of these methods.

To overcome this limitation, we need to explore comprehensive cfDNA fragmentation features from blood in the early events of tumorigenesis. Previous research studies have shown that variants in repetitive elements (REs) are associated with more than 50 serious human diseases (Hannan, 2018). These variants can specifically contribute to tumor development (Xing et al., 2019; Fujimoto et al., 2020; Hoyt et al., 2022), with a significant accumulation of alterations occurring in the initial phases of tumorigenesis (Hoyt et al., 2022). REs exhibit intricate biological functions that can catalyze genomic instability, thereby contributing to abnormal gene expressions or the emergence of pathogenic variants (Hannan, 2018; Helman et al., 2014). Recent studies have indicated that repetitive elements, such as Alu and short tandem repeats (STRs, also known as simple repeats), can impact enhancer–promoter interactions through amplification and indel events, leading to aberrant expression of anti-oncogenes and oncogenes (Shen et al., 2021; Liang et al., 2023; Jakubosky et al., 2020). Particularly, numerous studies have demonstrated that STR variants exhibit tumor-specific characteristics and affect the efficacy of immunotherapy in individuals with malignancies (Fujimoto et al., 2020; Erwin et al., 2023; Hause et al., 2016; Wooster et al., 1994). Hence, REs have great potential for cancer detection. In recent times, several studies have attempted to distinguish between healthy individuals and cancer patients by analyzing the ratio of different RE lengths in plasma (Sikora et al., 2015; Uto et al., 2016). Furthermore, a recent study has suggested that RNA fragments of REs in plasma have the potential to identify patients with cancer (Reggiardo et al., 2023). However, the potential of DNA-derived cell-free repetitive elements (cfREs) with more comprehensive fragmentomic profiles to identify cancers through whole genome sequencing (WGS) remains unknown.

In this study, we present our findings on the utility of fragmentomic profiles of cfREs (cfRE-F) for detecting multi-cancer through low-pass whole genome sequencing (lpWGS). There was a significant enrichment effect of cfREs, especially the Alu and STR elements, in the plasma of patients with cancer compared to that of healthy individuals. We performed a comprehensive analysis of five cfRE fragment profiles in plasma, namely, the fragment ratio (FR), fragment length (FL), fragment distribution (FD), fragment complexity (FC), and fragment expansion (FE). Furthermore, we built a multimodal approach based on machine learning to accurately identify multi-cancer across the sequencing depth. Through the characterization of fragments from tumor-specific enhancer and promoter regions within the cfRE, we were able to precisely localize the tissue of origin (TOO) and properly identify the aberrant transcription of cancer driver genes. Taken together, our study provides a framework for analyzing the genomic signature of cfRE fragments in plasma that can be used for sensitive, robust, and cost-effective tumor detection and localization.

## 2 Methods

### 2.1 Patient cohorts and study design

This study included 32 healthy volunteers and 112 early-stage and operable cancer patients from the National Cancer Center/Cancer Hospital, Chinese Academy of Medical Sciences, and Peking Union Medical College. Healthy volunteers were recruited after routine physical checkups. In brief, blood samples were obtained from participants over the course of two years (2020 to 2022). The information on the participants is summarized in Additional File 1 in Supplementary Table S1.

The study followed the ICH-GCP guidelines. All participants signed the informed consent form. The external independent validation dataset was GSE71378 (Snyder et al., 2016), and it was downloaded from the GEO websites. The independent dataset excluded non-cancer patients, and pan-cancer patients were used for validating the cancer prediction efficacy, while patients with the same cancer type as the internal cohort were selected for the evaluation of TOO performance.

### 2.2 Sample collection and cfDNA extraction

Approximately 10 mL of peripheral blood was collected from the subject using a Cell-Free DNA BCT® (Streck, Cat: 230471). Samples were delivered to the laboratory within 72 h, and plasma isolation was subsequently performed. A volume of 4 mL of plasma was used to extract cfDNA using a plasma cfDNA purification kit (Concert, Cat: RC1101), following the manufacturer’s instructions. cfDNA quantification was performed using the Qubit Fluorometer (Thermo Fisher Scientific, Cat: Q33231).

### 2.3 Library construction and sequencing

The libraries were constructed using the KAPA Hyper Library Prep Kit (KAPA Biosystems, KK8504), according to the manufacturer’s instructions. The library phosphorylation process was then completed using phosphorylation primers, followed by library cyclization and DNB generation (MGI, Cat: 1000005662), and sequencing was performed on the MGISEQ-2000 platform in the PE100 mode with 30G per sample.

### 2.4 WGS data processing and quality control

Sequence quality filtering and adapter trimming of reads were processed using fastp (v0.12.4) (Chen et al., 2018) with default parameters. After adapter trimming, reads were aligned against the hg19 human reference genome using BWA-MEM 0.7.17 (Li and Durbin, 2009) with default parameters. PCR-duplicate fragments were removed using GATK 4.2.0 (McKenna et al., 2010). Reads were retained if they met the following criteria: a mapping quality score of 30 or greater, no supplementary alignment, not a PCR duplicate, and both ends uniquely mapped. Finally, the mean effective depth of all WGS samples was 11.3.

### 2.5 cfDNA covered repeat elements processing and filtrations

Annotation files of RepeatMasker were downloaded from https://repeatbrowser.ucsc.edu/data/(Fernandes et al., 2020). Qualified mapped fragments were intersected with RepeatMasker genome locations using BEDTools (v2.31.0) (Quinlan and Hall, 2010). These overlapped regions were defined as cfDNA repeat elements and were filtered according to the following steps:

i) regions that could not be classified;
ii) regions located within the Duke blacklisted regions or sex chromosomes (http://hgdownload.cse.ucsc.edu/goldenpath/hg19/encodeDCC/wgEncodeMapability/);
iii) regions that covered zero fragments in more than 80% of the samples within the discovery cohort;
iv) regions that covered zero fragments in the GSM1833219 (mixed healthy human blood plasma) dataset (Snyder et al., 2016); and
v) repeat families with a genome-wide occurrence frequency of fewer than 500 instances.

### 2.6 cfRE signature definition and calculation

To analyze the cfRE, we designed five variables that illustrate the different patterns of cfREs between healthy and cancer disease samples: i) cfRE fragment ratio (FR); ii) cfRE short/long fragment length (FL); iii) cfRE non-zero covered ratio (FD); iv) cfRE reads complexity score (FC); and v) cfRE STR expansion score (FE).

The FR score was defined as the fraction of fragments mapped to the cfRE relative to the total number of qualified mapped fragments. The FL score was calculated as the ratio of the number of short fragments (fragment length less than 150 bp) to the number of long fragments (fragment length greater than 150 bp) within the cfRE. The FD score was the fraction of non-zero-covered cfREs within its family regions. The FC score was calculated as the median linguistic sequence complexity of the reads mapped to the cfRE.

We introduced a novel concept, the FE score, which represents the expansion factor of STR families. If the genomic position of an STR element is defined as chr-start-end, the repeat pattern is as follows:

$$\begin{array}{c} \underbrace{\left(\left[ATCG\right]..\left[ATCG\right]\right)}\\ n_{1} \end{array}\left[n_{2}\right],$$

where $n_{1}$ represents the repeat unit length and $n_{2}$ represents the number of times the unit is repeated. If the number of tandem repeat units covered by a read exceeded the reference $n_{2}$ , N_ETR (the number of expanded repeat units relative to the reference) was accumulated. Eventually, the single FE score was calculated within sub-groups for individual STR elements using the formula below:

$${Score}_{i}=\frac{{N\_ETR}_{i}}{N_{i}},$$

where ${N\_ETR}_{i}$ represents the number of reads containing unexpectedly expanded repeat units and $N_{i}$ is the total number of reads in each sub-group.

The STR family contains a large number of members, and to determine the weight of each member, we use the variable importance scores from a random forest (RF) model with 5-fold cross-validation on the training set; these scores were used to assign the weights to each STR sub-group. The FE score for each sample was calculated using the following equation:

$${FE}_{score}=\sum_{n=1}^{546}W_{i}{Score}_{i}.$$

### 2.7 Modeling and performance evaluation

After generating the cfRE signature input matrix of all samples, the datasets were split into the training set (98, 68%) and the test set (46, 32%) based on the sample enrollment date. The cancer prediction model was constructed by performing 10-fold cross-validation and using the algorithm of logistic regression with a LASSO penalty. The threshold of the model score was determined using the value corresponding to the Youden Index point. To determine whether the model performance could be preserved at low depths, we used Seqtk (v1.2-r101c) (Jeon et al., 2023) to randomly resample all samples with a gradient of simulated depth by 0.1×, 0.3×, 1×, 3×, and 5×.

### 2.8 Tissues-of-origin predictions using the cfDNA repeat element score

Only tumor patients were considered in the tissues-of-origin prediction. We downloaded the histone modification ChIP-seq wiggle files of H3K27ac/H3K4me1/H3K4me3 from six datasets corresponding to five cancer types [GSE136888 (Orouji et al., 2022), GSE76153 (Ooi et al., 2016), GSE212342 (Jeon et al., 2023), GSE67471 (Chen et al., 2015), GSE193257 (Gogleva et al., 2022), and GSE64557 (Diaferia et al., 2016)]: lung cancer, colorectal cancer, pancreatic cancer, liver cancer, and gastric cancer. The consensus signal peak region of histone modification intersected with the genome position of cfRE Simple_Repeat and cfRE Alu. The candidate TOO cfRE region needed to meet the following criteria: i) one type of modification peak occurred in more than half of the samples of corresponding ChIP-seq datasets; and ii) one candidate regulation cfRE region occurred in two or three types of histone modifications. Then, the cancer type-specific cfREs were determined using Fisher’s exact test, which computes the odds ratio and significance of cancer type preference. Finally, we calculated the FR, FL, FD, and FC scores of STR/Alu elements specific to each cancer type cfRE and built a multi-class logistic regression model for TOO prediction.

### 2.9 Statistical analyses

All statistical analyses were performed using R version 3.6.3. All the two-group comparisons were computed for p-value using the Wilcox-test. Based on true positive (TP), true negative (TN), false positive (FP), and false negative (FN) results of cancer prediction, we calculated the sensitivity [TP/(TP + FN)], specificity [TN/(TN + FP)], positive predictive value (PPV) [TP/(TP + FP)], negative predictive values (NPV) [TN/(TN + FN)], and accuracy [(TP + TN)/(TP + FP + TN + FN)]. The R package caret (v.6.0-79) (Kuhn, 2008) was used to implement the classification of healthy *versus* cancer samples and the tissue of origin. ROC curve and model output were obtained using the pROC (v.1.13) R package (Robin et al., 2011). The R package clusterProfiler (v4.2.2) (Yu et al., 2012) was used to carry out GO enrichment analysis. Pathway networks were computed and plotted using aPEAR (R package, v1.0.0) (Kerseviciute and Gordevicius, 2023).

## 3 Results

### 3.1 Characterization of cfREs in plasma using lpWGS

**FIGURE 1:**
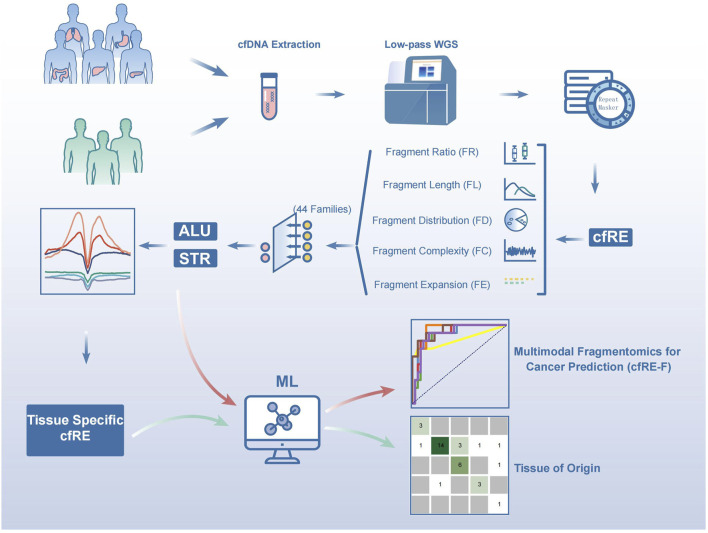
Schematic diagram of the workflow for early tumor detection by cfREs. Blood samples were collected from cancer patients and healthy controls, and low-depth whole genome sequencing was performed on plasma cfDNA. The qualified mapped reads were filtered and retained if they intersected with the RepeatMasker annotations. Five variables were designed according to the fragmentomic features of the repeat elements, and the most relevant Alu and STR families were screened out to establish a cancer prediction model. Cancer species-specific cfREs determined by histone modification were used to develop the model for predicting the origin of tumor tissue.

**FIGURE 2:**
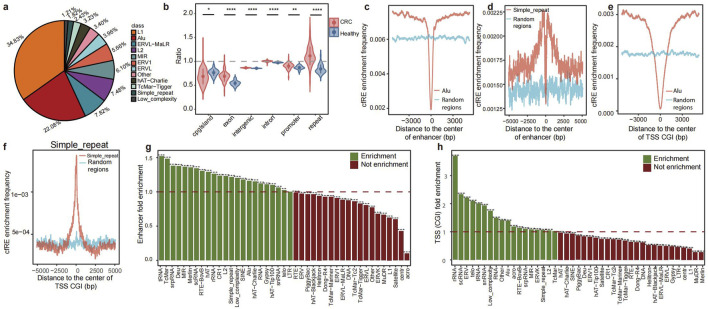
Characteristics of cfREs across the genome and pattern comparison between CRC patients and healthy controls. **(a)** Proportion of each repetitive element family in cfREs. **(b)** Comparison of the proportion of REs in the genome between CRC patients and healthy controls (significance levels for *p*-values are as follows: ns, p>=0.05; *, p < 0.05; **, p < 0.01; ***, p < 0.001; and ****,p < 0.0001). Distribution of Alu **(c, e)** and STR **(d, f)** family position distance from enhancer and TSS in the cfRE of CRC patients. Distribution of STR family positions from enhancer and TSS in the cfRE of CRC patients and healthy human controls. The enrichment of the 44 repetitive element family at enhancers **(g)** and promoters **(h)**; the y-axis represents the odds ratio of CRC patients compared to healthy controls.

We provided a biological approach to investigate the fragmentation characteristics of repetitive elements released into the plasma (Figure 1). This study involved four different datasets, which were obtained through lpWGS of cfDNA. These datasets were as follows: i) pilot cohort: this cohort consisted of three healthy individuals and three patients with colorectal cancer (CRC). Each dataset was created by merging five samples from the discovery cohort, resulting in an average raw depth of 50x; ii) Discovery cohort: this cohort included 76 patients with 5 types of cancer and 22 healthy individuals; iii) Validation cohort: this cohort included data from 10 healthy individuals and 36 patients with various types of cancer. The enrolled samples in the two cohorts had an average data depth of 10x (Supplementary Table S1); and iv) GSE71378 was used as the external validation dataset (Snyder et al., 2016). To identify cfREs, we conducted a comprehensive screening procedure using RepeatMasker (detailed in the “Methods” section). As a result, we identified a total of 37 cfRE families in the genome that are prone to being released into the plasma. Among these families, L1 and Alu were found to be more prevalent, while simple repeat and low-complexity repeat regions constituted a smaller portion (Figure 2a).

Next, we examined the distribution patterns of cfREs throughout the genome and compared their fragmentation patterns between healthy controls and patients with cancer. Previous studies have shown that REs are closely associated with transcriptional regulation and that cfREs are predominantly located in transcriptionally active chromatin regions (Figure 2b) (Criscione et al., 2014). We evaluated the characterization of all cfRE families across various transcriptional elements. The cfRE families enriched in enhancers, TSSs with CpG islands (TSS CGI), and other regulatory regions are mainly Alu, STR, and low-complexity-related families (Figures 2c–h). In conclusion, these results suggested that cfREs were involved in important biological functions, especially Alu and STR, and that there existed distinct fragmentation patterns between healthy individuals and cancer patients.

### 3.2 Fragmentomic profiles of cfREs effectively distinguish early-stage cancer patients from healthy individuals

**FIGURE 3:**
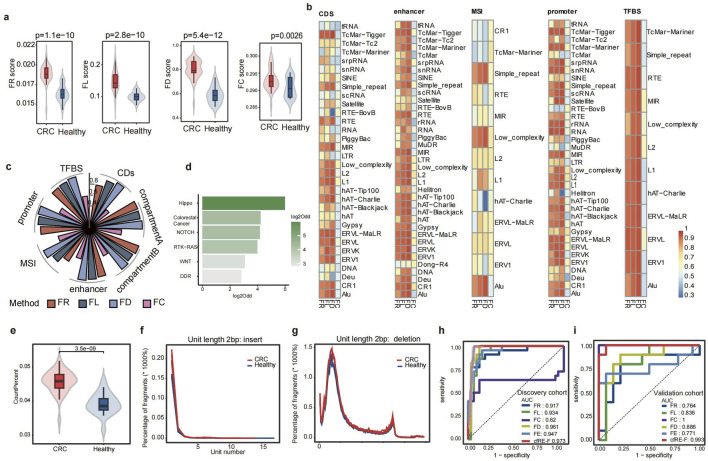
Fragmentomic characterization of cfRE and performance of the cfRE-F model. **(a)** Comparison of four cfRE fragmentomics variables between CRC and healthy controls in the discovery cohort. **(b)** Four variables from different subfamilies to distinguish CRC patients from healthy individuals after considering subsets of different functional regions in the human genome, and the color in the grid represents the AUC. **(c)** Performance of the four representative variables in predicting CRC *versus* healthy individuals in terms of different functional regions. **(d)** Pathways enrichment of regulatory regions corresponding to significantly altered cfREs in CRC patients, and the odds ratio was calculated for CRC cfRE *versus* random background. **(e)** Comparison of the proportion of STR-derived cfDNA fragments in CRC patients and healthy controls. Distribution of structural variation events of insertions **(f)** and deletions **(g)** of STR with 2 bp repeat units compared between CRC patients and healthy controls. The performance of the cfRE fragmentomics variables and the integrated CFR-F model for predicting CRC in the training **(h)** and validation **(i)** sets.

We analyzed the various fragmentomic profiles of all cfRE families to identify early-stage cancer patients. To assess the enrichment of fragments in the cfRE, we analyzed the proportion of fragments covering the region of each family in the whole genome (fragment ratio, FR). Similarly, to evaluate element activity, we calculated the proportion of regions with a fragment distribution for each family over all regions of the cfRE (fragment distribution, FD). Fragmentation lengths were profiled as the ratios of short (<150 bp) to long (≥150 bp) fragments for each family (fragment length, FL). Based on these features, there were significant differences between healthy individuals and CRC patients in the discovery cohort (Figure 3a). To investigate the impact of biofunctional regions, we performed an analysis of fragment characteristics in several functional domains. We assessed cfRE regions associated with different biological functions, such as compartment A/B (Fortin and Hansen, 2015), promoter, enhancer (Andersson et al., 2014), and coding sequence (CDS). The area under the curve (AUC) was compared for each family using four different scores to identify CRC in healthy individuals (Figure 3b). The correlation coefficients among the top 30% of families were consistently close to 1 (data not shown) based on the AUC ranking of the families. Notably, Alu and STR outperformed other elements in most features and functional classes (Figure 3b). To study the effect of biofunctional regions on repeat, we performed the AUC of CRC patients and healthy individuals within the specific domains. The FD score had the highest AUC compared to that of the other groups, with AUC values of 0.96, 0.96, 0.94, 0.96, 0.97, and 0.96 in compartments A/B, enhancer, MSI, promoter, TFBS, and CDS, respectively. Nevertheless, there was only a slight variation in AUC between the seven different functional components for four types of biomarkers (Figure 3c).

We next investigated whether differential cfRE could respond to aberrations involving gene regulatory elements in early-stage cancer. To further investigate the molecular mechanisms underlying these regulatory element abnormalities, we filtered the regions with a |z-score| >1 and an adjusted *p*-value <0.05 and performed gene annotation at the promoter (within the 1 kb upstream region of the TSS). Pathway enrichment analysis was performed on the annotated genes. As a result, we identified significant enrichment in two pathways: the Hippo pathway (log2Odd = 6), which regulates cell growth, proliferation, apoptosis, and tissue repair through the interaction of various signaling molecules (Sanchez-Vega et al., 2018); and the colorectal cancer pathway (Figure 3d). In summary, our findings suggested that cfREs exhibited overall aberrations in fragment patterns, and the pool singles may originate from small portions of the tumor tissue. These aberrations appeared to be closely associated with alterations in regulatory elements and pathways involved in carcinogenesis.

### 3.3 STR-specific fragmentation features improve the detection of cancer

STRs are usually regions of DNA repeats in the genome consisting of 1–6 bp units. Their high variability provided a more comprehensive characterization of the fragment. The 2 bp unit length elements are most prevalent in the STR regions, followed by the 4 bp unit length repeats (Supplementary Figure S1). STRs were classified into six groups based on their repeat unit lengths, ranging from 1 bp to 6 bp, and most STR repeat regions in these six groups were between 20 bp and 200 bp (Supplementary Figure S1). Except for the two groups with unit lengths of 2 bp and 3 bp, the number of repeat units in the other four groups is mostly 6–50 times (Supplementary Figure S1). Finally, we summarized a total of 546 sub-families based on the unit base length and repeat length of the STR in the plasma. This study showed that tumor patients were enriched with more fragments in the STR region (p = 3.5e^−09^) (Figure 3e), which is consistent with previous results (Erwin et al., 2023). To evaluate indels of STR fragments in the plasma, we analyzed the unit number variations of all 1–6 bp elements from the pilot cohort. We observed a tendency for the unit number of STR elements to differ between healthy individuals and patients with cancer in cfREs (Figure 3f,g; Supplementary Figure S2). In summary, fragments with more unit repeat counts than the reference genome were more active in tumor plasma, which is consistent with previous tissue-related studies.

It is well-known that the variants generated by STR elements promote tumor development and possess tumor specificity, so we need to further analyze the characteristics of STR fragments to improve tumor prediction performance. Inserting duplicate regions by unit is called expansion. We calculated fragment expansion (FE) based on the number of unit insertion reads detected in plasma, weighting each subfamily accordingly (details are provided in the “Methods” section). We distinguished between healthy individuals and CRC patients in the discovery and validation cohorts based on the STR expansion score (FE), and the AUC value was 0.947 and 0.771 in both cohorts (Figure 3h,i), respectively. To eliminate the effect of different cfRE families, we calculated the five signatures using only Alu and STR. The assay showed that the AUC for differentiating healthy individuals from CRC patients did not change significantly when reducing the families to Alu and STR only (Figures 3c,h,i). Therefore, only two families, Alu and STR, were selected for all subsequent features calculation. We first used the least absolute shrinkage and selection operator (LASSO) algorithm (10-fold cross-validation) to build a linear model based on five fragment properties in the discovery cohort and finally confirmed the model in the independent validation cohort. The AUC values of cfRE-F were 0.973 and 0.993 in two different cohorts (Figure 3h,i). The results showed that the FE score is complementary to the other features and also proved that the cfRE-F model outperformed models using a single indicator variable.

### 3.4 cfRE-F aids the detection of multiple early-stage cancers

**FIGURE 4:**
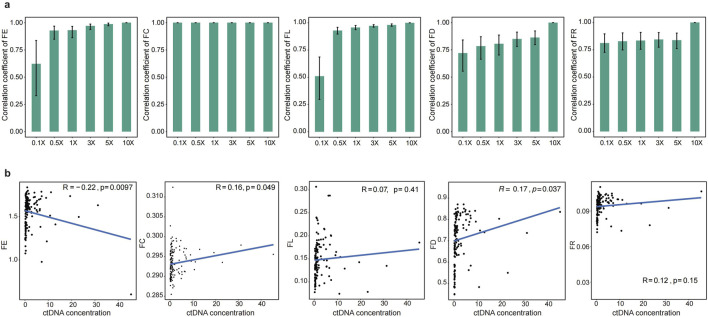
Performance of cfREs in predicting CRC at simulated low depths. **(a)** Prediction performance of the five cfRE variables at the simulated gradient random-sampling depth, which shows that the efficiency at 0.5× has been basically saturated. **(b)** Spearman correlation between the concentration of ctDNA and five cfRE variables in 10× depth.

To effectively address clinical needs, the cfRE-F solution should be expanded to incorporate multi-tumor assays and remain cost-effective. To this end, we would need to evaluate the performance of each characteristic at resampling depths. We randomly resampled from 0.1× to 10× for 10 times of healthy human D1 to simulate different sequencing depths. As we expected, the correlation coefficients of the values of the different indicators with the raw depths increased with increasing reads (Figure 4a). All the features are saturated at 0.5× depth, while the FC, FD, and FR scores are saturated when using 0.1× data, and the median correlation coefficients of these three measures with the original data are 1.0, 0.73, and 0.81 (Figure 4a), respectively. In conclusion, our study has shown that the cfRE-based fragmentation features can accurately restore the original signal in the ultra-low depth detection of WGS. In addition, we analyzed the correlation between fragment features and tumor DNA concentration. The absolute value of the correlation coefficient R between four fragment features and DNA concentration at sequencing 10× data was less than 0.3 (Pearson’s correlation coefficient, Figure 4b). Our analysis revealed that there was no correlation between four fragment features and DNA concentration with varying coverage depth.

**FIGURE 5:**
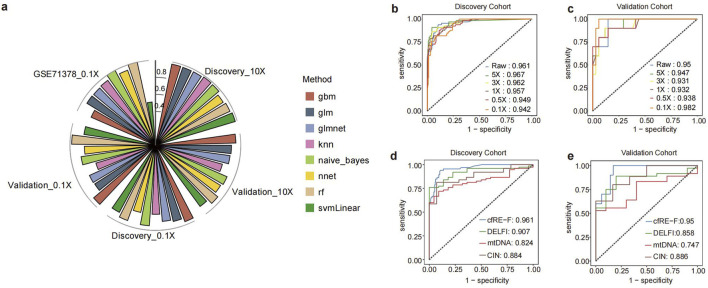
Determination of the CFR-F model building approach and comparison of its performance with other models. **(a)** Polar bar plot of performance comparison of cfRE-F models built using different algorithms in each dataset. ROC curves of cfRE-F in the training **(b)** and validation **(c)** sets at different depths. Comparison of the performance of cfRE-F with other early cancer detection models in the training set **(d)** and the validation set **(e)**.

We built a multiple feature-based ensemble machine learning model for cancer detection. For comparison, we tested eight different machine learning algorithms for the integrated modeling of five individuals based on all samples. The performance of the model was calculated using 10-fold cross-validation, and the results showed that the RF algorithm performed better in both the discovery and validation cohorts, with AUC values of 0.961 and 0.95 in raw depth (Figure 5a; Supplementary Tables S2-S4), respectively. To further test the reliability of the cfRE-F, we used the independent external validation set GSE71378 to predict pan-cancer (with nine types of cancers) from healthy individuals. To ensure unbiased modeling, we downsampled three different datasets to the same depth (0.1×). The AUC of the cfRE-F model based on the RF algorithm for the discovery, validation, and external validation sets were 0.95, 0.98, and 1 (Figure 5a), respectively. The results showed that the multimodal cfRE-F was effective for the prediction of tumors *versus* healthy individuals and was not limited to any cancer type.

To assess the cost-effectiveness of cfRE-F, we analyzed the detection performance from various sequencing analyses. We performed downsampling for all samples and then calculated the AUC changes at each depth based on cfRE-F. The analysis suggested that the discovery cohort had less change in AUC across reads, with AUC values of 0.961, 0.967, 0.962, 0.957, 0.949, and 0.942 from 10× to 0.1× (Figure 5b). Similarly, the AUC values for detecting patients with cancer in the validation cohort were 0.95, 0.947, 0.931, 0.932, 0.938, and 0.982 (Figure 5c). Meanwhile, we compared cfRE-F with DELFI, CIN, and mtDNA (Cristiano et al., 2019) at the same sequencing depth and found that cfRE-F outperformed the others (Figure 5d,e; Supplementary Figure S3). In conclusion, cfRE-F not only demonstrated the ability to detect multi-tumor types at an early stage but also showed ultra-sensitive and stable performance at low depths.

### 3.5 Fragmented cfRE profiles of regulatory element regions for multi-cancer localization

**FIGURE 6:**
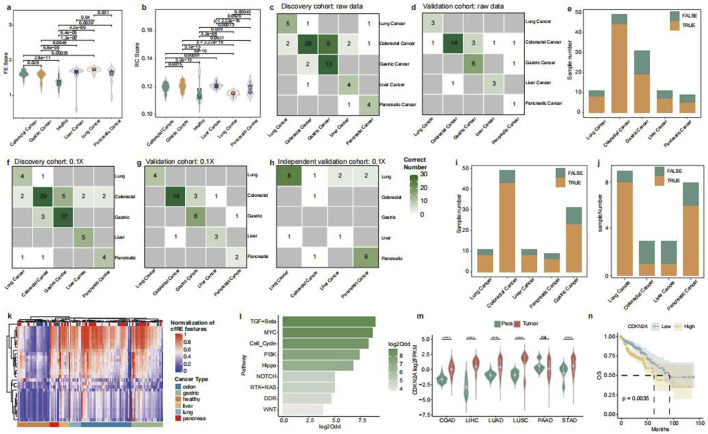
Model building of cfRE-TOO and genome enrichment of cancer-species-specific cfRE. Comparison of fragmentomics variables FE **(a)** and FC **(b)** for cancer-specific cfRE across cancer types. The correlation between true tumor location and cfRE-TOO predicted the tumor location in the discovery **(c)** and validation **(d)** datasets. **(e)** cfRE-TOO overall accuracy of prediction between different cancer types. The correlation between cfRE-TOO predicted and true source tumor locations for discovery **(f)**, validation **(g)**, and independent validation **(h)** datasets at an ultra-low depth of 0.1×. Accuracy of cfRE-TOO predictions in the validation **(i)** dataset and the independent validation **(j)** dataset at ultra-low depths. **(k)** Clustering of cfRE-TOO input variables, where different cancer types can be clustered in corresponding one class. **(l)** Tumor-specific cfRE relative genes enriched in tumor-associated pathways, with odds ratio computed in random gene background. **(m)** CDKN2A, as a tumor-specific cfRE-regulated gene, shows upregulated expression in different cancer types (significance levels for *p*-values are: ns, *p*>=0.05; *, *p* < 0.05; **, *p* < 0.01; ***, *p* < 0.001; and ****, *p* < 0.0001), and its high expression is a poor prognostic factor **(n)** for worse overall survival.

Alu and STR elements are tumor tissue-specific in aberrant transcriptional regulation. However, there may be a loss of information due to extremely limited sequencing depths. In recent years, it has been found that enhancers and promoters abnormally promote tumor development, and Alu and STR mainly mediate the recognition between enhancers and promoters. Therefore, we collected enhancer and promoter histone data of H3K4me1, H3K27ac, and H3K4me3 from colorectal, lung, gastric, liver, esophageal, and pancreatic cancers. This study enrolled six ChIP-seq datasets from five cancer types, including GSE136888, GSE76153, GSE212342, GSE67471, GSE193257, and GSE64557. Finally, we merged the identified tumor tissue-specific histone regions with the Alu and STR regions of the cfRE, and the overlapping regions were generated as the final tumor tissue-specific cfRE transcriptional regulatory element regions. A total of 85,498 regions were identified (Supplementary Table S5). We calculated different fragment signatures based on the tumor-regulated specific cfRE regions of Alu and STR, respectively. The FE score was identified as a specific marker for lung cancer, showing significantly higher values in lung cancer patients than in other tumor types and in healthy individuals (Figure 6a). The FC score calculated based on STR can effectively discriminate gastrointestinal tumors from liver (p = 0.00091) and lung cancers (p = 5e-16) (Figure 6b). All these different metrics were effective in distinguishing different tumors (Supplementary Figure S4). In conclusion, fragment features in tumor-specific cfRE regions based on regulatory elements could enhance the identification of different tissues of origin.

In order to trace the tumor-origin tissues, we developed a multi-tumor of origin classifier (cfRE-TOO), in which a prediction model was constructed based on the RF algorithm using five different fragmentation features of the tumor-specific cfRE with Alu and STR regions. The model correctly predicted tumor samples in both the discovery cohort 56/76 (73.7%) (Figure 6c) and the validation cohort 27/35 (77.1%) (Figure 6d), but P159 was excluded due to insufficiently calculated cfRE regions (Supplementary Table S6). The overall internal data accuracy was 74.1% (Figure 6e). We discovered that some misclassifications were assigned to highly correlated tissues. For instance, colorectal cancer was more likely to be predicted as stomach cancer. To assess the model’s robustness in ultra-low-pass scenes, we downsampled the data of the three cohorts to 0.1×. We then validated the accuracy of the model by constructing the cfRE-TOO model in discovery, followed by validation and external independent validation sets. The model correctly predicted 88/111 (79.3%) samples for the internal dataset (Figures 6f, g, i) and 16/23 (69.6%) samples for the independent external validation set (Figure 6h, j).

To further understand the biological functions of the tumor tissue-specific selected repetitive elements, we calculated five different indicators for specific regions across tumor types and illustrated the results in a heatmap (Figure 6k; Supplementary Table S7). Meanwhile, we performed enrichment analysis by selecting genes whose elements were in the promoter region and located in the tumor-associated pathway. The results showed that TGF-beta, MYC, and Cell_Cycle pathways were significantly enriched (Figure 6l; Supplementary Table S8). Among them, the mutated CDKN2A has been reported to be associated with the progression of various tumors. Our analysis of TCGA data revealed that it is barely expressed in normal tissues and is highly expressed in tumor tissues (Figure 6m). CDKN2A was associated with the prognosis of various tumors and is considered a relevant therapeutic target (Kreuger et al., 2023). A combined analysis of data from five different tumors in TCGA revealed that patients with low CDKN2A expression had a better prognosis, with hepatocellular carcinoma and lung squamous carcinoma reaching significant levels (Figure 6n; Supplementary Figure S5). In conclusion, tumor-specific transcriptional regulatory elements can be utilized to identify the origin of tumor tissues and tumor-specific prognostic markers and therapeutic targets.

## 4 Discussion

In this study, we reveal the feasibility of cfREs for the detection of multiple cancers by low-throughput whole genome sequencing. Regulatory elements have been reported to associate with aberrantly activated or switched-on chromatin during tumorigenesis, and these aberrant regulatory elements often exert their functions through repetitive elements (Anwar et al., 2017; Lee et al., 2024). Tumor cell-derived cfDNA shows a non-random breakage pattern during nuclease digestion, which is influenced by the chromatin state (Lo et al., 2021; Snyder et al., 2016; Cristiano et al., 2019; Chabon et al., 2020; Ulz et al., 2016). Cancer patients’ cfDNA has more repetitive elements than normal cfDNA, including distinct cfRE profiles. Aberrant changes in these elements, such as activation or structural variations, may cause genomic instability in tumorigenesis. cfRE fragmentomic features offer new ways to study cancer and other diseases.

Among the families of repetitive elements that distinguish healthy individuals from tumor patients, Alu and STR were the most important contributors. Alu plays important roles in gene expression regulation, DNA replication and recombination, and genome evolution (Hormozdiari et al., 2011). STRs are highly polymorphic and are strongly associated with cancer. In tumor cells, STRs may be gained or lost at high frequency due to the functional disruption of DNA replication or repair (Chatterjee and Walker, 2017). To comprehensively characterize the fragmentomic profile of cfREs in the plasma of cancer patients, we designed variables corresponding to tumor-specific repeated sequence variation events, and these features also provide important clues for cancer diagnosis and localization.

Repetitive elements, especially transposon elements, are increasingly recognized as tumor-driving factors and biomarkers. A representative one is COMPLETE-seq, which detects the expression of repetitive elements in cfRNA (Reggiardo et al., 2023). Another recently released study focuses on the application of the K-mer landscape of repetitive sequences for early tumor screening (Annapragada et al., 2024). Compared with these research articles, we have focused more on the fragmentation changes in representative repeat elements Alu and STR under repeat patterns. In addition to considering the content in cfDNA and the underlying fragment length distribution, changes in sequence complexity and expansion coefficients that may occur as a result of structural variations in the repetitive elements were also addressed. By performing an in-depth analysis of fragments in the cancer-type-specific enhancer and promoter regions of the cfRE, we were able to precisely trace the tissue origin of tumors and accurately identify the aberrant transcription of cancer driver genes. The predictive performance based on the tumor-specific cfRE fragmentomics model remains robust under ultra-low depth sequencing, and the cancer species-specific regulatory site-based cfRE model has considerable potential for tissue tracing.

In addition, we performed data sampling from 10× to 0.1× and found that the performance of the multimodal model at 0.1× (AUC = 0.982) was not inferior to that at 10× (AUC = 0.95). The overall detection cost of each sample is approximately 100 CNY, which is comparable to the cost of a polymerase chain reaction (PCR)-based assay. Hence, ultra-low-depth WGS effectively balances accuracy and cost, which is conducive to clinical promotion.

In summary, our study constructs a comprehensive framework for analyzing the genomic characteristics of cfRE fragments in plasma, which reconciles the advantages of high sensitivity and robustness and is remarkably cost-effective. The main limitation of the current study is that the sample size is not large enough. Furthermore, whole genome bisulfite sequencing (WGBS) can retain the fragmentation histological information of the repetitive elements and additionally supply the epigenetic information. Future studies should consider using low-depth WGBS to replace WGS to continue to improve the prediction performance of cfRE. In screening for cancer-type-specific repetitive elements, CUT&tag of histone modifications, ATAC-seq, and RNA-seq from various tumor tissues can be integrated to accurately identify altered RE sites specific to each cancer type. Future studies with larger cohorts and more multi-omics approaches will address the current problem.

## 5 Conclusion

In this study, we profiled five representative repetitive features of cfREs and explored their application in early cancer detection through low-pass whole genome sequencing. Alu and STR were the representative repetitive element families that robustly predict cancer and healthy cases. Furthermore, we developed a multimodal machine learning approach based on cfRE fragmentomics that accurately detects multiple early-stage cancers across different sequencing depths. The analysis of cfREs in tumor-specific regulatory regions demonstrated excellent accuracy in predicting the tissue of origin and identifying tumor driver genes with aberrant transcription. We performed additional testing on a limited number of independent external validation datasets. Overall, our study presents an innovative, sensitive, and cost-effective method that utilizes cfRE fragmentomics for enhanced cancer detection and localization.

## Data Availability

The datasets presented in this study can be found in online repositories. The names of the repository/repositories and accession number(s) can be found in the article/Supplementary Material.

## Abbreviations

cfREs, cell-free repetitive elements; cfDNA, cell-free DNA; lpWGS, low-pass whole genome sequencing; WGS, whole genome sequencing; STR, short tandem repeats; Alu, Alu elements; AUC, area under the curve; ROC, receiver operating characteristic; TOO, tissue of origin; LASSO, least absolute shrinkage and selection operator; RF, random forest; DELFI, DNA evaluation of fragments for early interception; CIN, Chromosomal instability; WGBS, whole genome bisulfite sequencing; ATAC-seq, assay for transpose-accessible chromatin using sequencing; TCGA, The Cancer Genome Atlas; GO, Gene Ontology; TFBSs, transcription factor binding sites; TSSs, transcription start sites; CDS, coding DNA sequence; FR, fragment ratio; FD, fragment distribution; FL, fragment length; FE, fragment expansion; FC, fragment complexity.
