# Supplementary material for: Comprehensive fragmentation of cell-free repetitive DNA for enhanced cancer detection in plasma
Source: Front Cell Dev Biol. 2025 Jul 9;13:1630231. doi: 10.3389/fcell.2025.1630231 (PMC12283650; doi:10.3389/fcell.2025.1630231)
Supplement: Supplementary file 1 [file Supplementaryfile1.docx]

Supplementary Material

## Supplementary Figures


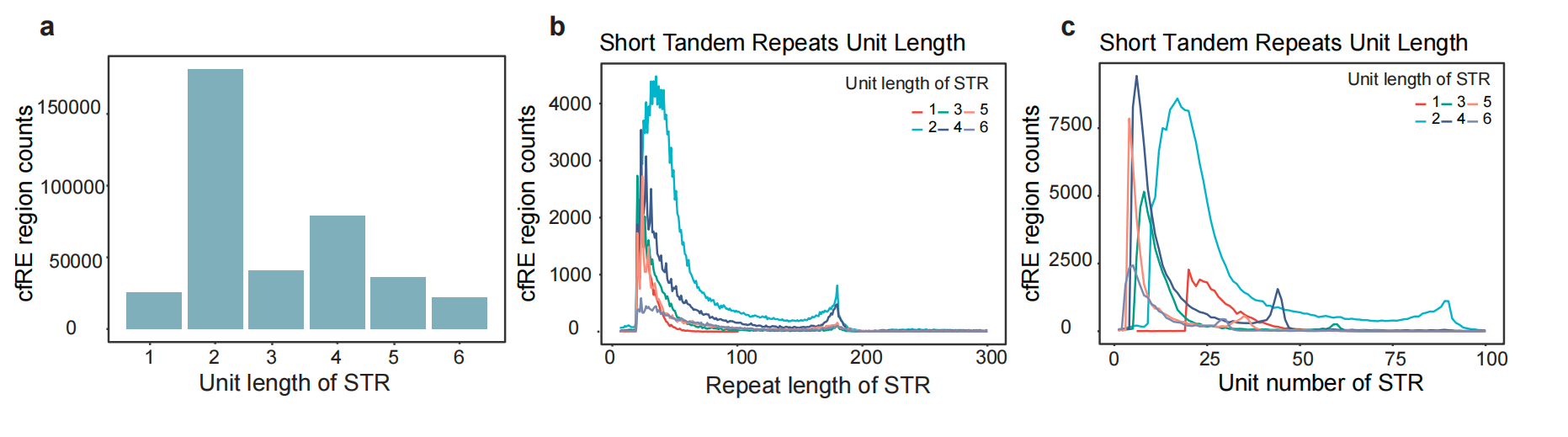


**Fig S1.** Distribution of STR constituent features (a) Unit length, (b) full length, (c) repeat times of unit.


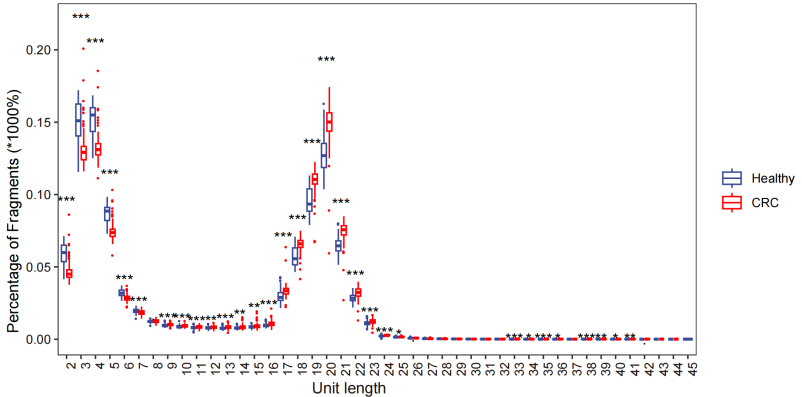


**Fig S2.** STR unit length comparison between Health and CRC.


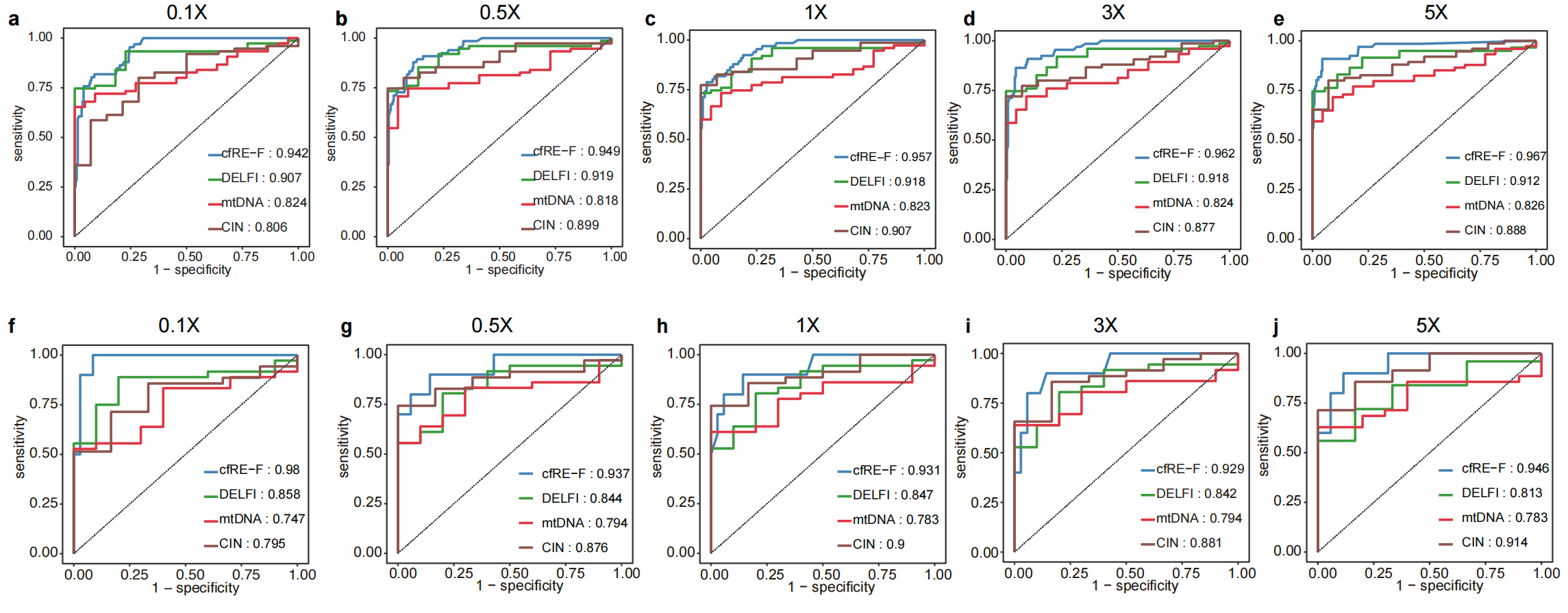


**Fig S3.** Comparison of the predictive performance of cfRE-F and other published cancer early screening algorithms at different depths in discovery (a~e) and validation (f~j) cohorts.


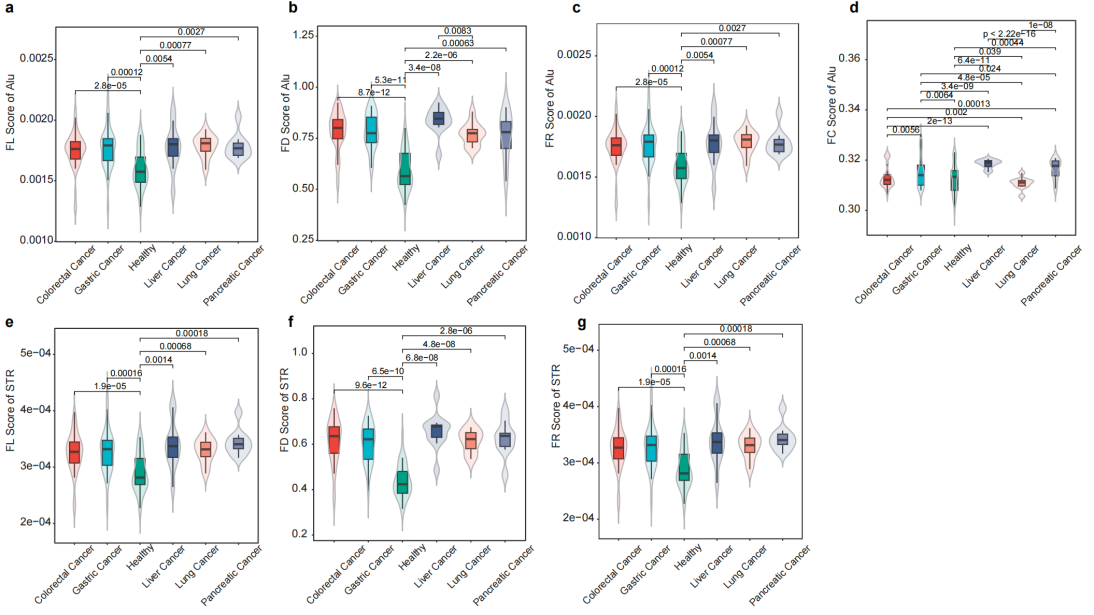


**Fig S4.** Comparison of four fragmentomics scores for ALU (a~d) and three fragmentomics scores for STR (e~g) among different cancer types.


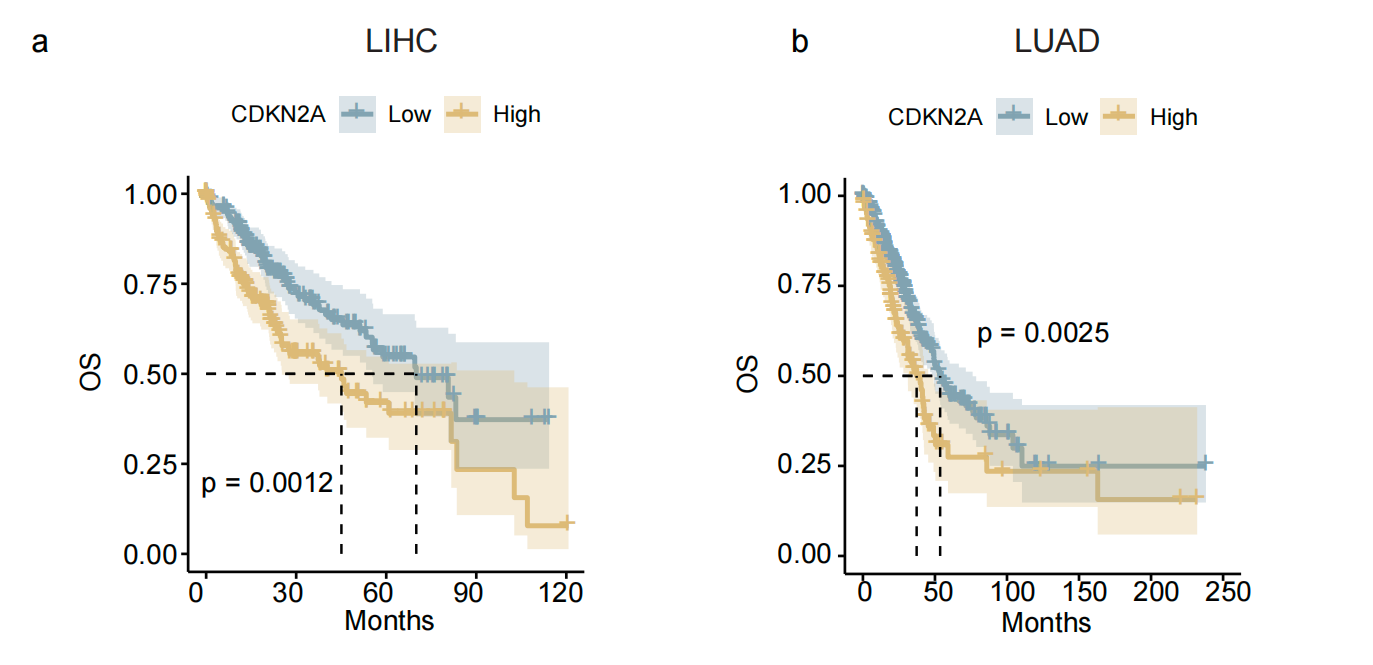


**Fig S5.** Representative genes related to repetitive elements CDKN2A, which with different expression levels have significantly different prognosis in TCGA LIHC (a) and LUAD (b).
